# Halofuginone Inhibits Osteoclastogenesis and Enhances Osteoblastogenesis by Regulating Th17/Treg Cell Balance in Multiple Myeloma Mice with Bone Lesions

**DOI:** 10.1007/s12288-024-01756-4

**Published:** 2024-03-25

**Authors:** Xiaofei Wu, Qiong Sun, Xiang Li, Lin Jiang, Li Chen

**Affiliations:** Department of Hematology, The Central Hospital of Wuhan, Tongji Medical College of Huazhong University of Science and Technology, Wuhan, 430014 China

**Keywords:** Multiple myeloma, Halofuginone, Osteoclast, Osteoblast, T cell

## Abstract

Evidences shows that T helper 17 (Th17) and regulatory T (Treg) cells imbalance plays a critical role in bone lesions of MM patients. Therefore, regulating the Th17/Treg imbalance may be beneficial for bone lesions in MM. Ten MM mice complicated with bone lesions were established and divided into the halofuginone (HF) group and the PBS group. After treatment, tibia and fibula from both groups were scanned by micro-CT. Osteoclasts and osteoblasts were validated by histochemical staining and ELISA. Th17 and Treg cells were tested by flow cytometry. The correlations between Th17/Treg cell ratio and osteoclasts, osteoblasts and bone remodeling were analyzed using the Spearman relative analysis. After treatment, mice in the HF group had an increase in trabecular bone volume fraction and thickened cortex, but a decrease in trabecular separation compared to mice in the PBS group.Tartrate-resistant acid phosphase (TRAP) + osteoclasts and its biomarker TRACP5b in serum were reduced, while alkaline phosphatase (ALP) + osteoblasts and its biomarker N-terminal propeptide of type 1precollagen (P1NP) in serum were accreted in the HF group. Th17/Treg cell ratio in halofuginone-treated mice was 0.85 ± 0.05, and was significantly lower than that in PBS-treated mice, which was 1.51 ± 0.03. In addition, it showed that the Th17/Treg cell ratio was significantly and positively associated with osteoclasts, but was significantly and negatively associated with osteoblasts and bone remodeling. Halofuginone plays a critical role in the amelioration bone lesions in MM, as it can inhibit osteoclastogenesis and enhance osteoblastogenesis by regulating the Th17/Treg cell balance.

**Supplementary Information:**

The online version contains supplementary material available at 10.1007/s12288-024-01756-4.

## Introduction

Multiple myeloma (MM), an incurable neoplasm with the second-most incidence in hematologic malignancy, is characterized by the malignant proliferation of monoclonal plasma cells in the bone marrow, and is frequently complicated with organ dysfunction, including bone lesion, anemia, hypercalcemia, and renal failure [1, 2]. Bone lesion is a main complication of MM which affects up to 80% of newly diagnosed patients, resulting in an increased risk of skeletal-related events, and more than a half of patients may experience bone fractures and need for surgical or radiotherapeutic treatment [3]. The mechanism of MM-related bone lesions has been well studied, of which the osteocyte-osteoclast-osteoblast axis disruption is considered to be the main reason leading to the formation of pathognomonic osteolytic lesions [4]. The microenvironment of bone marrow in MM is always interacted with myeloma cells, further drives osteolysis by promoting biochemical markers categorized as osteoclast-activating factors and osteoblast-inhibiting factors to release [5]. Elevated levels of osteoclast activating factors such as receptor activators of nuclear factor kappa B (RANK)/RANK ligand (RANKL)/osteoprotegerin (OPG) signaling, macrophage inflammatory protein (MIP)-1-α, tumor necrosis factor (TNF)-α, interleukin (IL)-3, IL-6 and IL-11 increase osteoclasts stimulation, differentiation, and maturation, whereas osteoblast inhibitory factors such as the Wnt/dickkopf1 (DKK1) pathway, peroxisome proliferator-activated receptor γ (PPAR-γ), and runt-related transcription factor 2 (Runx2) inhibit osteoblasts differentiation and formation leading to bone lesions [6, 7].

T helper 17 (Th17) and regulatory T (Treg) cells are major subtypes of naive CD4-positive (CD4^+^) T cells [8]. Th17 cells can induce the expression of IL-17 to protect host against fungal and parasitic infections, as well as participate in inflammatory and autoimmunity [9]. Evidence shows that Th17 cells and IL-17 expression have a significant increase in the bone marrow of MM patients compared to healthy persons [10]. Th17 cells and IL-17 also play a critical role in the microenvironment of bone marrow in MM patients, as they not only stimulate the growth of myeloma cells, but also break the normal bone balance and accelerate bone destruction by stimulating osteoclasts and inhibiting osteoblasts [11, 12]. Treg cells have immunosuppressive functions to prevent excessive damage by the immune system [13]. They can prevent myeloma cells dissemination, inhibit the stimulation and differentiation of osteoclasts by suppressing RANKL and macrophage colony-stimulating factor (M-CSF), and enhance the proliferation and differentiation of osteoblasts by secreting transforming growth factor (TGF)-β and activating mitogen activated protein kinase (MAPK), leading to an increase in bone mass [8, 14]. Considering the effects of Th17 and Treg cells on bone loss, we may conclude that Th17 cells can promote bone resorption while Treg cells can inhibit bone resorption. Therefore, by regulating the cross-talk between the Th17/Treg cell balance and bone cells, we may find new approaches for the treatment of MM. In accordance with above findings, Ren et al. [15] reported that the traditional Chinese medicine, Yunnan Baiyao, can significantly ameliorate rheumatoid arthritis in rats by decreasing Th17/Treg cell ratio and inhibiting the RANKL-induced osteoclasts formation.

Halofuginone is a small-molecular natural plant alkaloid and halogenated derivative of febrifugine which is originally extracted from a Chinese herb medicine, Dichroa febrifuga. It is well-known for the usage of antimalarial parasite for centuries, and has gained much attention because of its health-promoting properties in some diseases. Wang et al. [16] found that halofuginone significantly suppressed some pro-inflammatory cytokines' expression including IL-1β, IL-6, and TNF-α in vivo, as well as blocked the Th17 cell differentiation in vivo and vitro. It was confirmed by another study carried out by Mi-Kyung Park and colleagues [17], in which they reported that halofuginone reduced Th17 production and affected Th17 differentiation in autoimmune arthritis mice; furthermore, they also found that halofuginone can prevent the formation and activity of osteoclasts. In a MM-related study, Leiba et al. [18] claimed that halofuginone can suppress proliferation and induce apoptosis of MM cells. However, the effect of halofuginone on bone lesions has never been reported. Here, we report a bone lesions of MM mouse model study with the null hypothesis that halofuginone can ameliorate bone lesions by regulating Th17/Treg cell balance. The primary objective of this study was to evaluate the effects of halofuginone on bone lesion in MM mice. Secondary objective included the alterations of osteoclastogenesis and osteoblastogenesis, the changes of Th17/Treg cell ratio, and the mechanism of halofuginone's effect on bone lesions.

## Materials and Methods

### Animals

Ten male mice on C57BL/KaLwRij background at 7 weeks old were purchased from animal model center of Henan Skbex Biology Company, China. The mice were maintained in a specific pathogen-free environment under climate-controlled conditions (22 ~ 26℃ and 50% ~ 60% humidity) with a 12 h-light/dark cycle at Wuhan Central Hospital. After adaptively fed for a week, C57BL/KaLwRij mice were constructed to multiple myeloma model. They were irradiated for 300 cGy and injected intravenously with mouse 5TGM1 (1 × 10^7^ cells per mouse) cells. Two weeks later, they were divided into experimental group (HF group) and controlled group (PBS group) randomly, each group contained 5 animals. Mice in HF group were treated with 1 mg/kg halofuginone (MedChemExpress, USA) for three time a week, and mice in controlled group were treated with phosphate-buffered saline (PBS) (Solarbio, China) for vehicle control with a same frequency. All mice were euthanized after three weeks of treatment.

All experimental protocols were approved by the ethical committee of Wuhan Central Hospital (WHZXKYL2022-006) and were conducted in accordance with the China institutional guidelines for the care and use of animals.

### Micro Computed Tomography

Mouse tibiae and fibula were dissected and fixed in 10% neutral-buffer formalin for 2 days. μCT of tibia and fibula were performed using VivaCT80 scanner (Scanco, Switzerland). Scans were acquired at 10.4 μm isotropic voxel size, 55 kV,145μA, 200 ms integration time for a 360-degree scan with a 0.24-degree rotation. After scanning, images were reconstructed using the Scanco software, and the Otsu’s thresholding method was utilized to segment bone from non-bone [19]. Measurements performed to segment cortical and trabecular bone using the Dragonfly’s Bone Analysis included trabecular bone volume fraction (bone volume/total volume, BV/TV, %), trabecular thickness (Tb.Th, mm), trabecular separation (Tb.Sp, mm), Trabecular number (Tb.N, 1/mm), and cortical thickness (Ct.Th, mm).

### Tartrate-Resistant Acid Phosphatase (TRAP) and Alkaline Phosphatase (ALP) Staining

Tibiae were fixed in 10% formalin for 24 h then stored in 70% ethanol. After decalcification and paraffin embedding, 3 μm longitudinal sections of tibia were taken out for TRAP and ALP staining to identify osteoclastogenesis and osteoblastogenesis according to manufacturers’ instructions (Beyotime, China).

### Enzyme-Linked Immunosorbent Assay (ELISA)

Blood was centrifuged for 10 min to collect plasma. ELISA kit (Biowamp, China) was used to detect plasma levels of IgG2b, tartrate-resistant acid phosphatase isoform-5b (TRACP5b) and procollagen 1 intact N-terminal propeptide (P1NP).

### Flow Cytometry

Mice bone marrow T lymphocytes were washed with PBS to adjust the cell concentration to 1 × 10^6^ cells/ml after activated with PMA (20 ng/ml, Merck, Germany) and ionomycin (2 μg/ml, Merck, Germany), in the presence of monensin sodium (3 μg/ml, Sigma, USA). For Th17 cells assay, 100 μl of spleen T lymphocyte suspension was placed in a Falcon tube, then added FITC-conjugated anti-CD4 (Invitrogen, USA), then added PE-conjugated anti-IL-17A (Invitrogen, USA), incubated for 40 min at 4℃ in the dark. Then cells resuspended in 400 μl PBS were added, and the flow cytometer NovoCyte (ACEA Biosciences, China) were used to detected Th17 cells. For Treg cells assay, 100 μl of spleen T lymphocyte suspension was placed in a Falcon tube, then added FITC-conjugated anti-CD4 and PE-Cyanine5-conjugated anti-CD25 (Invitrogen, USA), then added PE-conjugated FOXP3 (Invitrogen, USA) monoclonal antibodies, incubated for 40 min at 4℃ in the dark. Then cells resuspended in 400 μl PBS were added, and the flow cytometer NovoCyte were used to detected Treg cells.

### Statistics Analysis

The experimental data were analyzed using GraphPad Prism 8 (GraphPad Software, USA). All data were presented as mean ± standard deviation (SD). Results between the PBS group and the HF group were compared using an unpaired two-tailed Student’s t test, one-way ANOVA, or two-way ANOVA with Tukey’s multiple comparisons test. The correlation between Th17/Treg cell ratio and TRACP5b, P1NP, BV/TV, Tb.N, Ct.Th were analyzed using the Spearman relative analysis. A two-tailed *P* < 0.05 was considered to be statistically significant.

## Results

### Halofuginone Ameliorates MM Bone Lesions and Attenuates Tumor Burden in Mice

Halofuginone ameliorates MM bone lesions and attenuates tumor burden in mice.

**Fig. 1:**
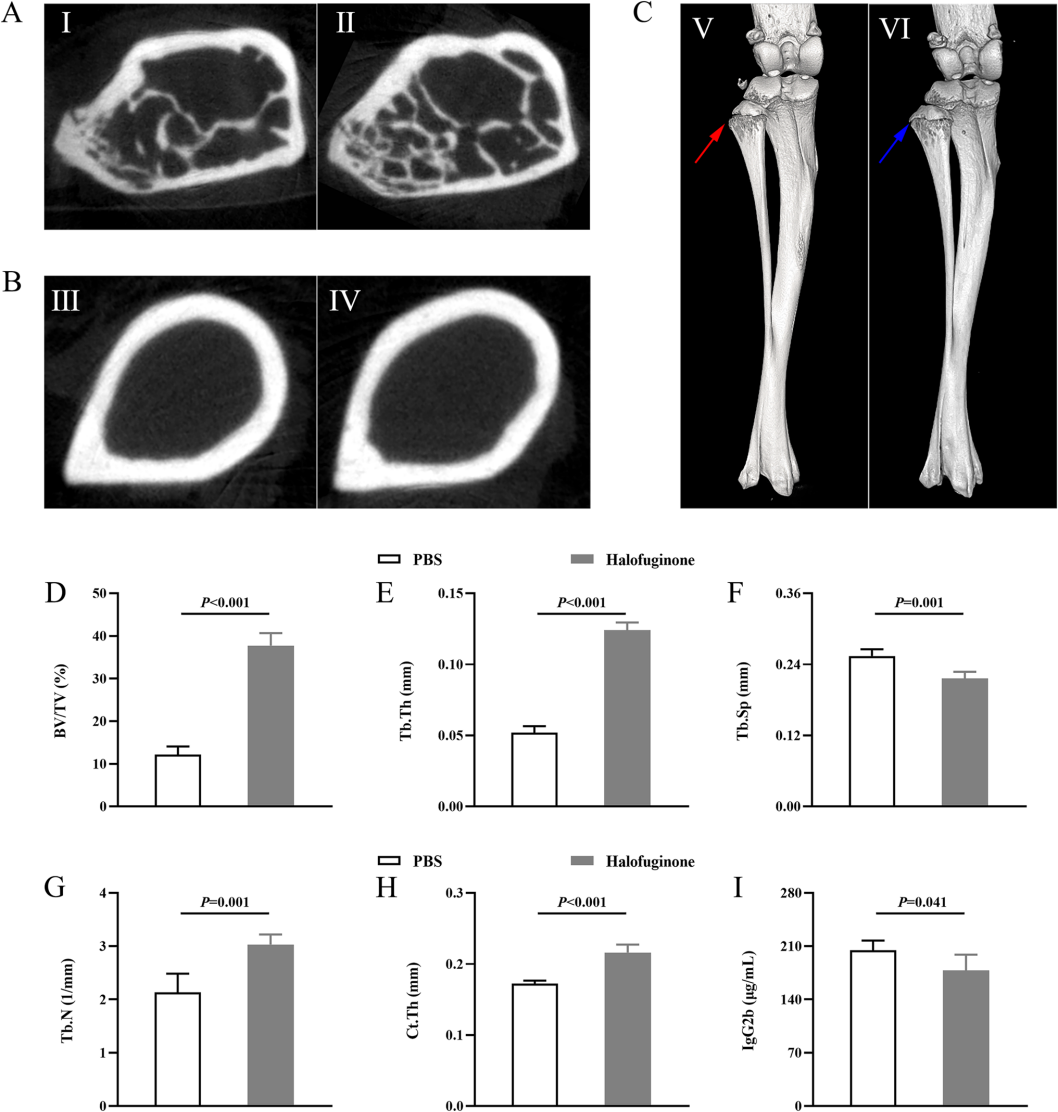
Halofuginone ameliorates MM bone lesion and attenuates tumor burden in mice. **A** 2D cross-sectional μCT images of tibial trabecular bone in the PBS and HF group. **B** 2D cross-section μCT images of tibial cortical bone in the PBS and HF group. **C** Overview of VOI selection in tibia (red arrow refers to bone lesion in the PBS group and blue arrow refers to bone repair in the HF group). **D**-**K** Comparisons of BV/TV, Tb.Th, Tb.Sp, Tb.N, Ct.Th and IgG2b between the PBS and HF group

At the endpoint of treatment, mice in the HF group (Fig. 1A-II) had an increase in trabecular bone volume fraction and a decrease in trabecular separation compared to mice in the PBS group (Fig. 1A-I). As shown in Fig. 1D and 1F, BV/TV was 37.73% (SD: 2.91) in the HF group, and was significantly higher than that in the PBS group of 12.15% (SD: 1.92); Tb.Sp was 0.21 mm (SD: 0.01) in the HF group, and was significantly lower than that in the PBS group of 0.25 mm (SD: 0.01). At the same time, thickened cortex of bone (Fig. 1B-II) and a higher Ct.Th (0.21 ± 0.01 mm) (Fig. 1H) was also found in halofuginone-treated mice. In consistent with the unimproved bone lesions in the PBS group (Fig. 1C-I), bone remodeling was induced and bone lesions were ameliorated in the HF group (Fig. 1C-II). Other indexes of bone remodeling like Tb.Th and Tb.N were also significantly different between two groups, with the Tb.Th and Tb.N were 0.12 mm (SD: 0.00) and 3.03 1/mm (SD: 0.19) in the HF group but were 0.05 mm (SD: 0.00) and 2.13 1/mm (SD: 0.35) in the PBS group (Fig. 1E, G). In addition, monoclonal globulin IgG2b in the halofuginone-treated mice was 177.95 pg/mL (SD: 18.70), and was significantly lower than that in the PBS-treated mice, which was 207.21 pg/mL (SD: 15.31) (Fig. 1I).

### Halofuginone Inhibits Osteoclastogenesis and Enhances Osteoblastogenesis

**Fig. 2:**
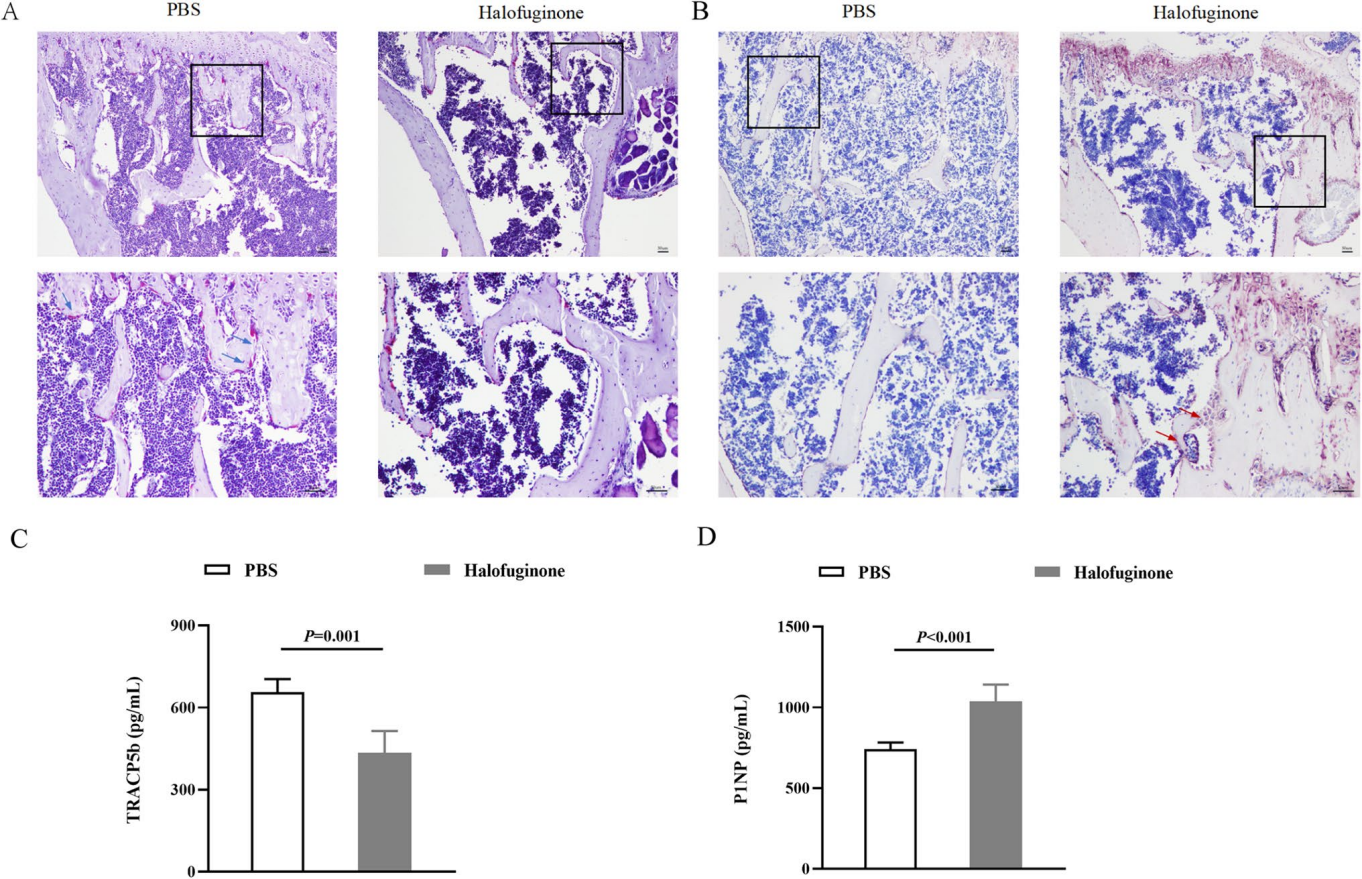
Halofuginone inhibits osteoclastogenesis and enhances osteoblastogenesis. **A** TRAP-stained osteoclasts in tibia of the PBS group and HF group (signed by red arrow). **B** ALP-stained osteoblasts in tibia of the PBS group and HF group (signed by blue arrow). **C**-**D** serum TRACP5b and P1NP levels in the PBS and HF group

After staining, TRAP positive osteoclasts present as red and ALP positive osteoblasts present as brown. As shown in Fig. 2A, at the endpoint of treatment, osteoclast count in tibia remained large in the PBS group but obviously decreased in the HF group. Those changes in osteoclasts were supported by serum TRACP5b (Fig. 2C), as the serum TRACP5b concentration was 656.32 pg/mL (SD: 42.74) in PBS-treated mice, and was significantly higher than that in halofuginone-treated mice, with a concentration of 434.28 pg/mL (SD: 71.49). With regarding to osteoblastogenesis in tibia, osteoblast count in tibia remained low in the PBS group but obviously increased in the HF group (Fig. 2B). Similarly, those changes in osteoblasts were supported by serum P1NPb (Fig. 2D), as the serum P1NP concentration was 741.57 pg/mL (SD: 36.33) in PBS-treated mice, and was significantly lower than that in halofuginone-treated mice, with a concentration of 1038.11 pg/mL (SD: 93.01).

### Halofuginone Regulates Th17/Treg Cell Imbalance

**Fig. 3:**
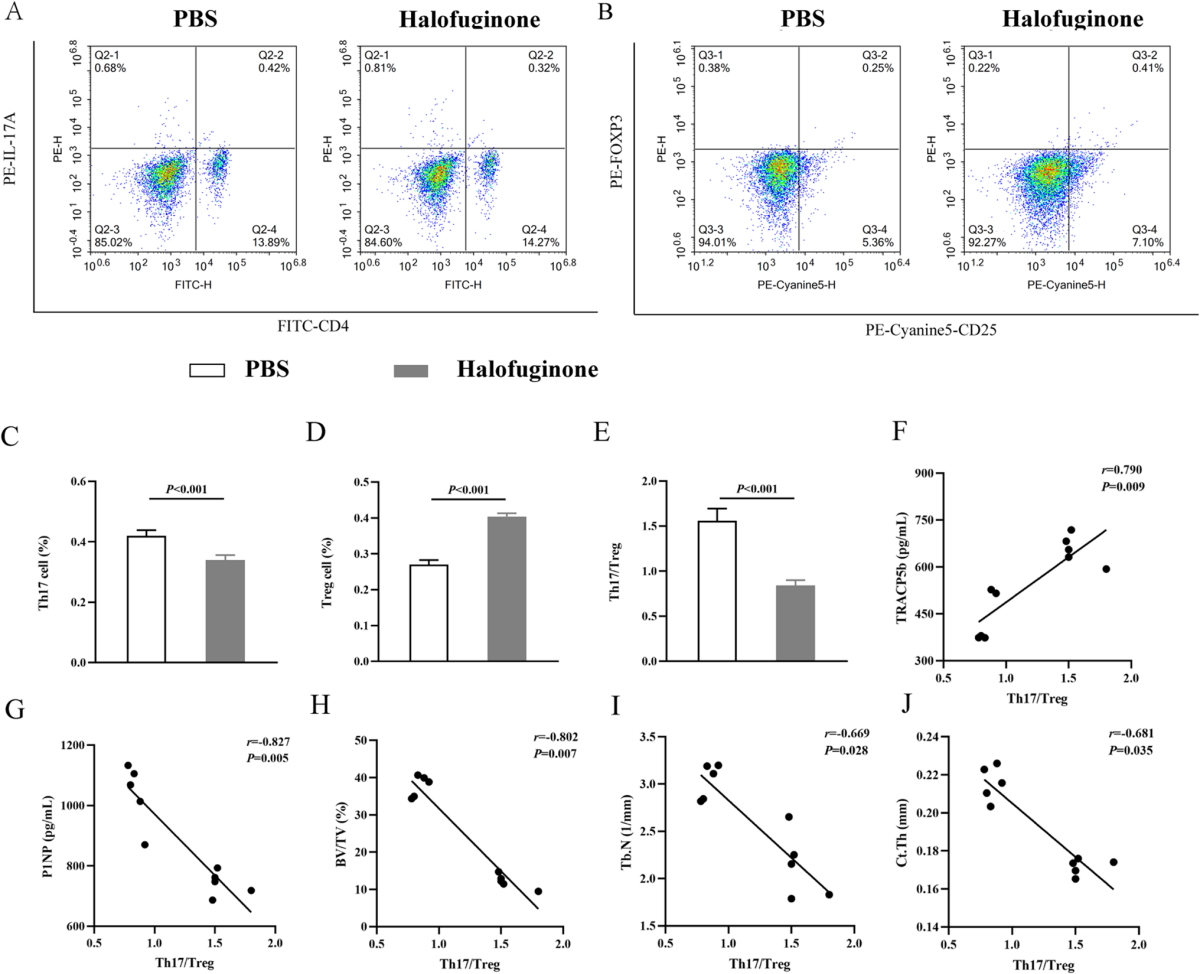
Halofuginone regulates Th17/Treg cell imbalance. **A** and **C** Th17 cell counts measured by flow cytometry and ELISA in the PBS and HF group. **B** and **D** Treg cell counts measured by flow cytometry and ELISA in the PBS and HF group. **E** Th17/Treg cells ratio in the PBS and HF group. **F**-**H** The association between Th17/Treg cell ratio and BV/TV, Tb.N, Ct.Th analyzed by linear regression analysis

To investigate the mechanism of halofuginone in bone lesion regulation, we detected Th17 and Treg cells in mice by flow cytometry. Firstly, Th17 and Treg cells of mice at the day before being constructed to MM model (0d, healthy mice) and two weeks later after being constructed to MM model (14d, MM mice) were measured. Compared to Th17 and Treg cells in healthy mice, Th17 cell count increased 50.0% but Treg cell count decreased 17.6% in MM mice (Fig. S1A-S1D). Accordingly, Th17/Treg cell ratio in MM mice was 0.37 ± 0.01, and was significantly higher than that in heathy mice, which was 0.21 ± 0.02 (Fig. S1E). At the endpoint of treatment, compared to Th17 and Treg cells in PBS-treated mice, Th17 cell count decreased 16.7% but Treg cell count increased 49.6% in halofuginone-treated mice (Fig. 3A-D). Accordingly, Th17/Treg cell ratio in halofuginone-treated mice was 0.85 ± 0.05, and was significantly lower than that in PBS-treated mice, which was 1.51 ± 0.03 (Fig. 3E). After treatment, we constructed Spearman relative analyses to analyze the correlation between Th17/Treg cell ratio and TRACP5b, P1NP, BV/TV, Tb.N, Ct.Th, results showed that the Th17/Treg cell ratio was significantly and positively associated with TRACP5b, while r was 0.790, and P value was 0.009; however, it was significantly and negatively associated with P1NP, BV/TV, Tb.N and Ct.Th, while r was -0.827, -0.802, -0.669, and -0.681, respectively, and all P values were below 0.05 (Fig. 3F-J).

## Discussion

In present study, we investigated the effects of halofuginone on bone lesions and its mechanism in MM mice. For the primary outcome, the bone lesions were remarkably ameliorated and the tumor burden was obviously attenuated by halofuginone. Our results also indicated that halofuginone can inhibit osteoclastogenesis, enhance osteoblastogenesis, and shift the Th17/Treg cell imbalance. Furthermore, the mechanism of halofuginone's effect on bone lesions may be that halofuginone inhibited osteoclastogenesis and enhanced osteoblastogenesis by shifting Th17/Treg cell imbalance, as results of Spearman relative analysis showed that the Th17/Treg cell ratio was significantly and positively associated with TRACP5b, but was significantly and negatively associated with P1NP, BV/TV, Tb.N and Ct.Th after treatment.

TRACP5b is a specific biomarker to osteoclasts and formed during osteoclast development [5, 20]. The serum level of TRACP5b can reflect osteoclast activity and differentiation directly. In current study, results showed that both the TRAP + osteoclasts and the serum TRACP5b concentration decreased obviously in the HF group, indicating that halofuginone reduced osteoclast formation and activity in MM. Similar results have been observed in autoimmune arthritis mice and chronic periodontitis mice when they were treated with halofuginone [16, 17]. However, in another study researchers found that when the doses of halofuginone were less than 10 μg/ml, no effect of halofuginone was evident on osteoclast abundance or morphology, reflecting that halofuginone had no direct effect on osteoclast formation, but was mediated by other factors [21]. P1NP is a byproduct of type I collagen synthesis and an indicator of bone formation and osteoblast function [22, 23], and its serum concentration can reflect osteoblast activity and differentiation directly. We found that both the ALP + osteoblasts and the serum P1NP concentration increased obviously in the HF group, suggesting that halofuginone enhanced osteoblastogenesis by increasing osteoblast formation and activity. As this finding has been never reported in previous studies, we are the first to investigate and report the effect of halofuginone on osteoblasts. Benefit from the inhibitory of osteoclastogenesis and the enhancement of osteoblastogenesis, bone lesions in MM mice were significantly ameliorated after halofuginone treatment.

After treatment, it showed that Th17 cell count decreased but Treg cell count increased in halofuginone-treated mice, and Th17/Treg cell imbalance was shifted, revealing that halofuginone can inhibit Th17 cells differentiation and stimulate Treg cells formation. Similar findings were observed when halofuginone was used to treat autoimmune encephalomyelitis mice [24], autoimmune thyroid disease mice [25], and severe acute hepatitis B virus (HBV)-infected SD rats [26], in which researchers found that halofuginone had obviously therapeutic efficacy on those diseases by reducing Th17 cell differentiation and/or increasing Treg cell formation. The potential mechanism of Th17/Treg cell balance regulation by halofuginone may be mainly caused by interleukins and TGF-β. Bettelli and colleagues found that in the presence of IL-6, IL-23 and TGF-β, IL-6 activated signal transducer and activator of transcriptions 3 (STAT3) to suppress the expression of forkhead box protein p3 (Foxp3), and upregulated IL-23 receptor expression to induce immature T cells to differentiate into Th17 cells; however, in the absence of IL-6 and other pro-inflammatory factors, TGF-β drove the differentiation of immature T cells into Treg cells [27].

Considering the effects of Th17 and Treg cells on osteoclast differentiation and osteoblast formation, we conducted a Spearman relative analysis to analyze the association between Th17/Treg cell ratio and osteoclastogenesis, osteoblastogenesis, and bone formations. Results showed that the Th17/Treg cell ratio was positively associated with osteoclasts, but was negatively associated with P1NP, BV/TV, Tb.N and Ct.Th. According to previous studies, the potential mechanism underlying Th17 cell-induced changes in osteoclasts and osteoblasts may involve pathogenic Th17 cells with high expression of RANKL regulating autophagic activity of osteoclasts through the RANKL-JNK1 signaling pathway, thereby enhancing osteoclast differentiation and reducing apoptosis [28, 29].Th17 enhance RANKL expression of osteoblasts by down-regulating autophagic activity through JAK2/STAT3 pathway [30]. Treg cells inhibit osteoclasts differentiation and maturation by upregulating the secretion of osteoprotegerin (OPG) and downregulating the expression of RANKL and M-CSF [31], whereas they enhance osteoblasts proliferation and differentiation by secreting TGF-β, activating intracellular effectors such as mitogen activated protein kinase (MAPK) and Smad-related proteins [8]. The results depicted in Fig. 2 demonstrated that halofuginone effectively inhibited osteoclast differentiation and activity, while simultaneously enhancing the differentiation and activity of osteoblasts, thereby significantly ameliorating bone lesions. Additionally, halofuginone administration leads to a reduction in the Th17/Treg cell ratio in MM mice with bone lesions. Considering the crucial role played by Th17/Treg cells in regulating osteoclasts and osteoblasts, these findings suggest that halofuginone improves bone lesion outcomes through modulation of the Th17/Treg cell balance. However, further investigation is required to elucidate the underlying molecular mechanisms.

The strength of the present study is that we are the first to evaluate the effects of halofuginone on bone lesions and its mechanism in MM mice, and results suggest that halofuginone can ameliorate bone lesions in MM based on osteoclastogenesis inhibitory and osteoblastogenesis enhancement, which may be mediated by regulating the Th17/Treg cell balance. However, the present study has several limitations. Firstly, MM mice used in this study were established by injecting mouse 5TGM1 cells, so the bone lesion and the follow-up halofuginone- and PBS-treated experiment were all based on mouse-MM mice. In fact, at the pre-experimental phase, we tried to established the human-MM mice by injected with human RPMI8226 cells. However, those human-MM mice cannot induce bone lesions, so we chose the mouse-MM mice with bone lesions to investigate the halofuginone’s effect on bone lesions, and we believed that the results still had reference value. Secondly, in this study we focused our attentions on the amelioration of bone lesions, changes of osteoclasts, osteoblasts, Th17 cells, and Treg cells, but lacked the attentions on variation tendency osteoclast and osteoblast pathways such as RANKL, NF-ATc1, and Wnt, so the molecular mechanisms cannot be completely analyzed.

In conclusion, this study revealed a critical role of halofuginone in the amelioration bone lesions in MM. halofuginone can inhibit osteoclastogenesis and enhance osteoblastogenesis by regulating the Th17/Treg cell balance, which are important to exert effector responses in plasma cell neoplasm. Understanding the mechanisms by which halofuginone regulates both T cell immune responses and bone systems have profound importance of potential therapeutic intervention in MM-related bone loss.

### Supplementary Information

Below is the link to the electronic supplementary material.

Supplementary file1 (TIF 1007 KB)
