# Supplementary figures and images for: Halofuginone Inhibits Osteoclastogenesis and Enhances Osteoblastogenesis by Regulating Th17/Treg Cell Balance in Multiple Myeloma Mice with Bone Lesions
Source: Indian J Hematol Blood Transfus. 2024 Mar 25;40(3):407–14. doi: 10.1007/s12288-024-01756-4 (PMC11246324; doi:10.1007/s12288-024-01756-4)

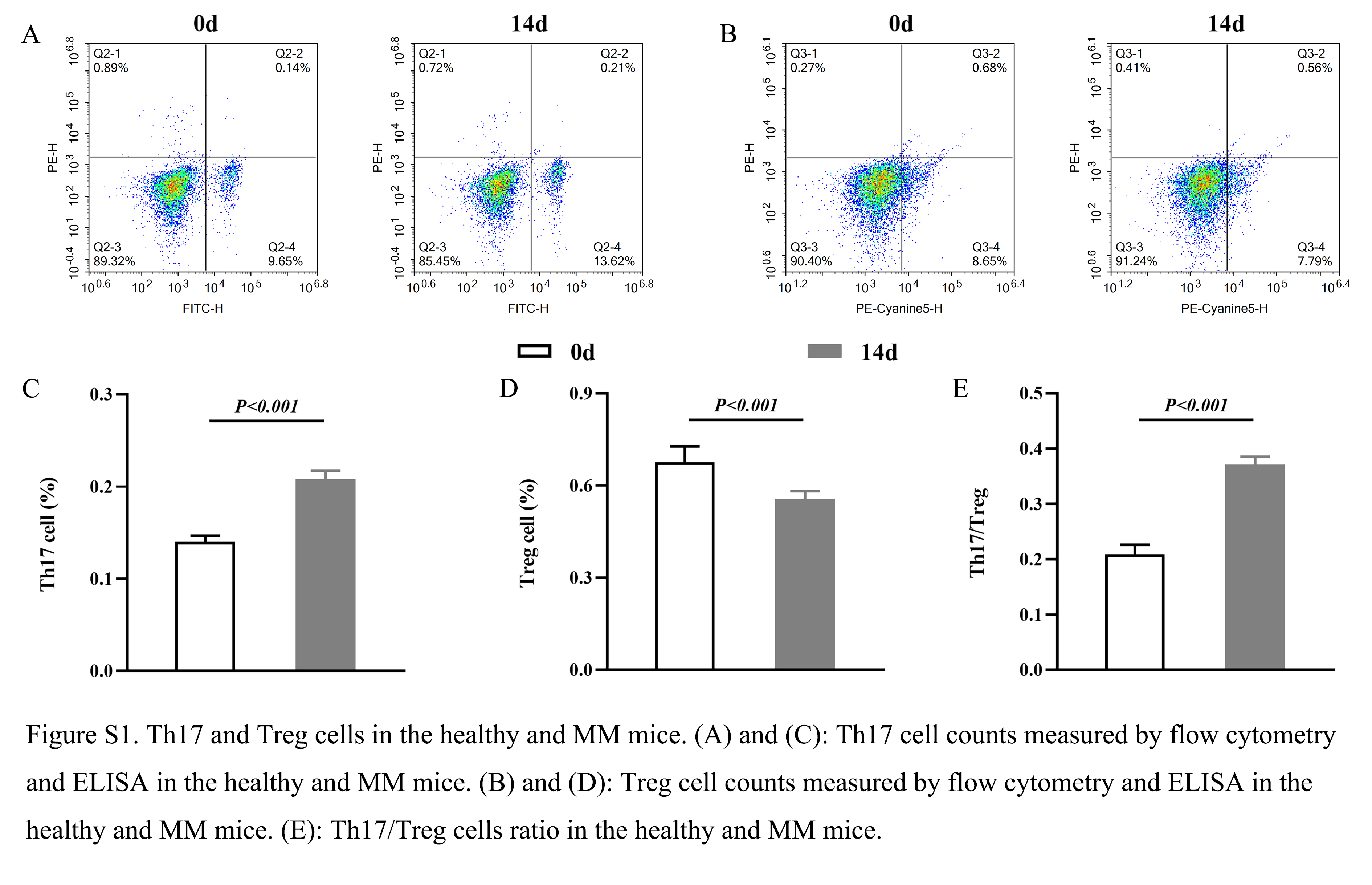

Supplement: Supplementary file 1 — Supplementary file1 (TIF 1007 KB) [file 12288_2024_1756_MOESM1_ESM.tif]
